# Supplementary material for: Healthcare Utilization for Lateral Epicondylitis: A 9-Year Analysis of the 2010–2018 Health Insurance Review and Assessment Service National Patient Sample Data
Source: Healthcare (Basel). 2022 Mar 28;10(4):636. doi: 10.3390/healthcare10040636 (PMC9030966; doi:10.3390/healthcare10040636)
Supplement: Supplementary file 1 [file healthcare-10-00636-s001.zip › healthcare-1641747-supplementary.pdf]

Table S1. Annual average KRW-USD exchange rate and price level of health expenditure.

Year	KRW/USD	Price level
2010	1,156.00	0.9345
2011	1,107.99	0.9510
2012	1,126.76	0.9597
2013	1,095.04	0.9631
2014	1,053.12	0.9699
2015	1,131.52	0.9820
2016	1,160.41	0.9918
2017	1,130.48	1.0005
**2018**	**1,100.58**	**1.0000**

This information is available on the following site: Korean Statistical Information Service (http://kosis.kr).

The price level of health expenditure is adjusted as of year 2018.

Table S2. Basic characteristics of medical usage.

Category		Total (2010-2018)		WM (2010-2018)		KM (2010-2018)	
		No. of claims	%	No. of claims	%	No. of claims	%
**Type of visit**	Outpatient	757,661	99.78	591,902	99.72	165,759	99.98
	Inpatient	1,686	0.22	1,652	0.28	34	0.02
**Medical institution**	Medical center†	110,437	14.54	110,266	18.58	171	0.1
	Medical clinic	482,957	63.6	482,957	81.37	-	-
	KM medical center‡	3,347	0.44	331	0.06	3,016	1.82
	KM clinic	162,606	21.41	-	-	162,606	98.08

WM, Western Medicine; KM, Korean Medicine.
All expenditures were converted with annual average exchange rate (KRW/USD) (See Supplementary Table 1)

†Claims in KM (n = 171): KM clinics within medical centers

‡Claims in WM (n = 331): WM clinics in KM medical centers

Table S3. Number of patients by month, year, and age in detail.

Month*	Age	Year								
		2010	2011	2012	2013	2014	2015	2016	2017	2018
1 (-1.29)	Under 15	6	3	3	4	4	6	4	1	2
	15-24	14	20	19	40	23	28	24	27	19
	25-34	69	67	77	93	96	94	77	72	90
	35-44	421	442	491	461	573	551	506	498	507
	45-54	756	880	955	1001	1095	1146	1182	1087	1217
	55-64	363	429	543	559	588	717	728	781	817
	65-74	159	125	174	209	226	256	227	242	243
	75 or older	30	26	42	41	47	50	52	43	70
2 (1.29)	Under 15	0	4	8	3	4	3	3	2	1
	15-24	13	15	23	22	19	22	29	23	14
	25-34	75	67	85	85	76	75	81	84	75
	35-44	421	436	515	494	480	502	512	511	486
	45-54	725	828	974	984	1029	968	1120	1121	1028
	55-64	339	386	497	534	581	576	683	785	747
	65-74	149	159	167	212	189	179	231	252	228
	75 or older	18	23	33	30	45	48	48	57	56
3 (6.49)	Under 15	5	10	8	3	12	6	11	7	8
	15-24	25	30	30	25	24	20	37	30	23
	25-34	89	86	93	86	92	107	80	94	103
	35-44	415	474	533	482	594	575	555	585	564
	45-54	806	937	974	989	1094	1121	1256	1189	1212
	55-64	395	455	531	521	593	669	755	799	917
	65-74	171	171	207	212	203	222	265	259	273
	75 or older	30	34	48	35	34	61	50	49	67
4 (12.34)	Under 15	5	12	5	4	4	5	8	6	9
	15-24	17	22	26	25	30	26	25	26	19
	25-34	97	82	74	93	99	93	84	66	88
	35-44	460	457	399	495	431	503	474	480	485
	45-54	790	802	796	869	899	1005	970	1048	1033
	55-64	338	408	441	501	505	570	602	666	691
	65-74	137	147	181	169	188	210	208	220	243
	75 or older	28	24	34	40	42	42	49	41	56
5 (18.06)	Under 15	7	6	5	8	3	7	2	8	11
	15-24	13	20	25	40	35	29	34	33	36
	25-34	76	79	69	89	87	83	92	94	74
	35-44	458	496	544	524	481	491	505	485	511
	45-54	745	851	876	997	904	919	1046	1040	1083

	55-64	321	355	488	536	565	563	622	671	730
	65-74	136	133	156	223	172	231	230	209	218
	75 or older	24	32	32	38	42	32	57	51	54
6 (22.09)	Under 15	12	6	7	10	4	10	13	10	4
	15-24	24	23	29	31	28	25	31	28	31
	25-34	83	84	86	83	81	73	103	92	78
	35-44	444	486	525	510	506	484	538	532	535
	45-54	725	831	890	862	978	991	1088	1068	1122
	55-64	334	399	433	486	578	532	619	716	755
	65-74	138	132	142	181	169	188	210	213	219
	75 or older	15	34	32	29	46	46	54	54	52
7 (25.6)	Under 15	6	3	5	5	6	6	6	5	2
	15-24	24	24	20	26	26	33	26	28	17
	25-34	84	58	76	83	83	68	95	85	89
	35-44	407	419	458	469	483	441	475	474	437
	45-54	704	746	776	925	918	951	936	993	1045
	55-64	324	349	414	456	482	530	595	666	636
	65-74	121	121	143	171	162	193	187	204	217
	75 or older	28	21	28	32	35	42	39	46	46
8 (26.02)	Under 15	5	6	6	8	6	9	2	5	6
	15-24	24	22	25	21	29	26	23	36	30
	25-34	72	110	113	89	86	108	105	84	85
	35-44	441	476	504	546	476	498	491	525	473
	45-54	767	866	923	988	953	939	1021	1103	1039
	55-64	323	383	466	506	494	544	633	644	691
	65-74	144	144	151	197	183	173	188	223	182
	75 or older	22	24	37	31	40	35	36	41	60
9 (20.92)	Under 15	7	4	12	9	8	9	8	9	6
	15-24	19	14	24	27	30	22	36	26	26
	25-34	67	76	72	73	91	86	95	100	85
	35-44	410	459	458	434	487	526	474	482	494
	45-54	643	759	776	758	916	981	997	1030	1020
	55-64	303	318	359	388	518	492	576	663	622
	65-74	105	133	139	141	170	155	180	217	191
	75 or older	17	21	29	24	32	42	38	44	62
10 (14.66)	Under 15	12	2	9	7	10	9	8	1	4
	15-24	16	16	24	27	22	27	37	24	31
	25-34	83	70	97	88	82	85	65	71	70
	35-44	343	430	430	456	458	451	420	431	472
	45-54	600	702	791	835	792	846	813	879	955

	55-64	258	325	369	452	430	469	492	541	605
	65-74	112	101	139	122	129	186	176	169	207
	75 or older	17	26	29	28	30	49	45	32	47
11 (8.16)	Under 15	8	6	11	6	5	5	3	2	7
	15-24	20	28	23	21	36	24	29	34	30
	25-34	77	90	96	83	97	118	99	95	75
	35-44	467	518	497	528	492	496	535	516	502
	45-54	823	931	901	952	878	947	1046	1091	1061
	55-64	344	404	417	505	474	567	612	664	719
	65-74	160	144	173	172	166	199	218	207	216
	75 or older	23	24	46	28	41	33	46	51	58
12 (0.94)	Under 15	9	7	13	6	3	8	3	5	7
	15-24	23	24	17	22	32	36	37	33	27
	25-34	100	122	95	119	105	121	136	107	118
	35-44	526	632	648	676	682	692	718	698	659
	45-54	1039	1131	1118	1230	1237	1264	1296	1390	1312
	55-64	431	490	521	653	677	787	869	895	899
	65-74	187	190	190	223	228	264	270	262	272
	75 or older	35	35	33	50	50	63	55	48	58

* 9-year average monthly temperature(°C) in parentheses. Monthly average temperature data is available on the following site: Korea Meteorological Administration Weather Data Service (https://data.kma.go.kr/)

Table S4. Within-hospital prescriptions.

Categories	No. of Claims	Average expenditure per claim	Average expenditure per patient
Anti-inflammatory enzymes	159,970	0.88	2
NSAIDs	306,352	2.51	6.42
Acetaminophen(AAP)	108,777	1.81	4.3
Antispasmodic	10,871	1.52	3.21
Propulsives	167,923	2.38	5.61
Other propulsives	104,893	1.21	2.77
Antacids	29,015	0.45	0.97
Psychiatric agents	10,625	0.73	1.6
Muscle relaxants	99,737	1.4	2.96
Hormones	19,724	0.36	0.73
Antibiotics	1,541	3.96	5.83
Blood circulation agents	8,071	4.98	10.59

All expenditures were converted with annual average exchange rate (KRW/USD) (See Supplementary Table 1)
